# The Physical Burden of Water Carrying and Women’s Psychosocial Well-Being: Evidence from Rural Nepal

**DOI:** 10.3390/ijerph18157908

**Published:** 2021-07-26

**Authors:** Vica Marie Jelena Tomberge, Janine Stefanie Bischof, Regula Meierhofer, Akina Shrestha, Jennifer Inauen

**Affiliations:** 1 Department of Health Psychology & Behavioral Medicine, Institute of Psychology, University of Bern, Fabrikstrasse 8, 3012 Bern, Switzerland; janine.bischof@psy.unibe.ch (J.S.B.); jennifer.inauen@psy.unibe.ch (J.I.); 2 Department of Sanitation, Water and Solid Waste for Development (Sandec), Eawag, Swiss Federal Institute of Aquatic Science and Technology, Ueberlandstrasse 133, 8600 Duebendorf, Switzerland; Regula.Meierhofer@eawag.ch; 3 School of Medical Sciences, Dhulikhel Hospital, Kathmandu University Hospital, Dhulikhel 1008, Nepal; akinakoju@gmail.com

**Keywords:** gender inequalities in health, water access, psychosocial well-being, unpaid work, low-income population

## Abstract

Many women in low-income countries carry heavy loads of drinking water for their families in difficult terrain. This can adversely affect their health and well-being. The present study is the first to investigate the physical burden of water carrying and women’s psychosocial well-being, and how this relationship is moderated by environmental and health conditions. Trained local interviewers conducted interviews with 1001 women across five rural communities in Nepal. In addition, objective measurement was used to assess the weight carried and distance from the water source. The physical burden of water carrying was calculated from weight, distance, and frequency of trips. Its association with psychosocial well-being was modeled using generalized estimating equations. Two additional models included the terrain and uterine prolapse as moderators. The physical burden of water carrying is directly related to higher emotional distress and reduced daily functioning. This correlation was exacerbated for women carrying in hilly versus flat terrain, and for those who had uterine prolapse. Our results underline the importance of adequate water access for women’s psychosocial well-being, especially for vulnerable populations such as women with impaired health (e.g., uterine prolapse) or those living in hilly terrain. The results further highlight the interconnectedness of the Sustainable Development Goal (SDG) 6: water access, SDG 3: health and well-being, and SDG 5: gender equality.

## 1. Introduction

Water is needed in many areas of life, such as drinking water, food production, care of domestic animals, hygiene, cleaning, and waste disposal [1]. In 2017, 25% of the global population collected water from sources that are located off premises [2]. Previous research on the health consequences of sub-optimal water access has described adverse impacts of low water quality [3,4], the transport of water [5,6,7] water insecurity [8,9], and poor menstrual hygiene management [10,11].

Traditionally, mostly women are responsible for collecting and providing water for their families and livestock [12,13]. Particularly in water poor areas, the time required to retrieve water can pose barriers to other activities, such as education, paid work, and healthcare, which results in impairment of women’s quality of life [14]. The responsibility of water collection can further pose a serious threat to women’s psychosocial well-being [15,16,17].

Psychosocial well-being is an integral part of health as defined by the World Health Organization (WHO) [18] and represents a multidimensional construct which incorporates emotional, social, and physical aspects [19]. Adverse psychosocial well-being has been associated with water insecurity [15,16,17], with findings implying that high water insecurity relates to increased emotional distress [15,17] and lower quality of life [16]. In addition, evidence from sub-Saharan Africa shows that the risk of sexual harassment and violence on the route to, or while queueing for, water can be a source of fear and stress [15]. The physical burden of carrying water may be an additional source of distress. However, studies on this are absent.

Research on carrying heavy loads in general supports a potential relationship between water carrying and psychosocial well-being [20,21]. Excessive occupational workload adversely affects a person’s emotional or physical energy and time available for other activities. This, in turn, is linked to increased psychosocial distress [22,23].

Social and cultural norms created a gendered division of labor in many developing countries, with women being primarily responsible for unpaid domestic work [24]. Domestic work is substantial to the functioning of the household. However, women are often not being acknowledged for their work [24]. Research suggests that a gendered division of housework is one of the factors that generally contributes to the differences between men and women in regard to adverse health effects, such as higher psychosocial distress for women [25]. A study in Ghana also showed that strenuous domestic work had adverse effects on women’s well-being [20]. Moreover, a study in Nepal showed that anxiety and depression were more prevalent among people who carried heavy loads [21]. In the context of water carrying, this could imply that high physical burden, due to frequently carrying heavy water containers, might relate to decreased psychosocial well-being.

Past research investigating the consequences of water carrying largely focused on physical health effects and disability [26]. They neglected context-specific conditions of water carrying that can pose an added risk to health [7]. For example, when water containers are carried in challenging or uneven terrain, the risk of falling and injury is high [7,13]. Hence, terrain could moderate the relationship between the physical burden of water carrying and psychosocial well-being.

Another challenge is that frequent carrying of heavy water containers exerts significant strain on women’s bodies [5]. This can lead to disabilities such as musculoskeletal disorders or uterine prolapse [5,6,27]. Uterine prolapse indicates that the uterus descends from its normal position into or out of the vagina [27]. Approximately 19% of women in low- and middle-income countries are affected by pelvic organ prolapse, which also includes uterine prolapse [27]. Uterine prolapse can severely affect women’s daily lives if they are unable to work, have difficulties standing up, walking or lifting heavy loads, and are subject to social stigmatisation within families and communities [28,29]. Moreover, uterine prolapse is associated with increased emotional distress and lower quality of life [29,30]. Irrespective of their health condition, e.g., uterine prolapse, women in rural areas are expected to complete their assigned household tasks such as fetching water [29,31,32]. Not being able to complete this responsibility, or having difficulties in doing so, is likely to put an extra burden on women affected by uterine prolapse [31].

### The Present Study

In the present study, we investigate the psychosocial consequences of water carrying for women. Specifically, we investigate whether higher physical burden of water carrying relates to lower psychosocial well-being, and whether terrain or having uterine prolapse moderate this relationship. We study this at the example of Nepal. In 2017, only 26% of the population in Nepal had access to an improved water source [33]. Due to the mountainous regions, women in rural Nepal have to walk on unpaved roads, often uphill while carrying water-filled containers [34]. At least 11% of women in the reproductive age in these areas have had uterine prolapse [35]. Based on present literature, we hypothesize that: (1) high-objective physical burden is related to lower psychosocial well-being; (2) carrying water in hilly terrain strengthens the relationship between water carrying and psychosocial well-being, compared to carrying on a flat path; (3) having uterine prolapse strengthens the relationship between water carrying and psychosocial well-being.

## 2. Materials and Methods

The present survey was conducted between September and November 2019 in five communities in the Kavre and Sindhupalanchowk district of Nepal. This is a typical rural low-income region with a mixture of water supply on and off premises, such as private taps in the court of houses, shared taps or surface water in neighborhoods, and community taps or surface water around villages. The study areas were selected because they included outreach centers of the Dhulikhel Hospital, our collaborator. Ethical clearance was given by the Ethical Review Committee of the Health Research Council Nepal [Reg No. 517/2019] and the Ethical Review Board of the University of Bern, Switzerland [2019-10-00003]. Study aims and procedure were explained to all participants in the local language. All participants provided written informed consent prior to the interview. Potential study participants who were unable to sign their names indicated consent with their thumbprint.

### 2.1. Survey Procedure and Participants

Leading to a total sample size of 1001 women, four trained local health scientists and four health practitioners selected approximately 200 women per community following the random route method [36]. The data collectors started from a central point in an assigned area of the community and assessed if there was an eligible person living in the approached household who consented to participate. It was predetermined whether either every second or third household in the community was approached, based on the total number of households in the community (every second household for smaller communities, every three households for larger communities). The selection criteria for respondents targeted adult women (from 16 years in Nepal) in reproductive age who permanently resided in the community, were involved in water carrying, and willingness to participate. If there was more than one woman within the household who met the selection criteria, the woman predominantly responsible for water carrying was interviewed. If no eligible person was available in that household, the data collectors assessed if there was an eligible person willing to participate in the household that was skipped before selecting the current household.

The survey consisted of a computer-assisted personal face-to-face interview and objective measurement of weight and distance. Health practitioners additionally carried out physical health examinations. When a participant indicated symptoms of uterine prolapse, a free screening at the local health centre was performed, and if necessary, free treatment was offered.

### 2.2. Measures

The survey instruments were translated and back-translated from English to Nepali and pretested in one community not included in the analyses. Please consult Table 1 for sample items and descriptives, and Table S1 in the Supplementary Material for all questionnaire items.

#### 2.2.1. Physical Burden

An adapted version of the risk assessment for lifting and carrying suggested by SUVA, the Swiss National Accident Insurance Fund [41] was applied to calculate the objective physical burden of water carrying. The adapted formula includes the following weighted risk variables: (weight + environmental condition) × (carrying frequency × distance).

We assessed weight for all water containers carried in one trip (verified with a scale) and distance from the house to the water source in meters. The distance was recorded with a GPS device (Garmin CSX 60). Frequency represents the self-reported number of trips a day. For the environmental condition, we decided to categorize all women to the second out of three SUVA risk categories which includes: “Stability restricted by uneven, soft ground”. The original formula presented by SUVA suggests also including body posture. As we wanted to focus on the burden of environmental factors only, we decided to exclude this variable for this analysis. Furthermore, we had many missings for this variable (*n* = 105).

Missings for observed weight were replaced by the average of self-reported minimum and maximum weight carried per trip, as perceived by the best guess [42]. Values were missing due to technical difficulties (no scale unit given by interviewer, *n* = 76) or because of missing information on carrying behavior due to current health conditions (*n* = 34). To avoid bias in the statistical models, prior to modeling, outliers in weight were adapted to *M* + 3 *SD* (55.8 kg) [40]. Missings for distance (*n* = 21) were not replaced. These adaptions resulted in a sample size of *n* = 978, which is 98% of the overall sample and likely inconsequential for biases and loss of power [43]. Physical burden was thus used as a continuous variable with higher scores representing higher burden.

#### 2.2.2. Psychosocial Well-Being

To assess psychosocial well-being we included measures of emotional distress (WHO’s SRQ-20, 36), quality of life (WHOQOL-12, 37) and one item on daily functioning (Functioning rating scale, 38). Emotional distress (α = 0.81) consists of somatic, depressive/anxiety, and cognitive/decreased energy symptoms [44]. Quality of life assesses the domains of physical, psychological, social, and environmental well-being [38]. The goal to use all 26 items of the quality of life scale was deemed too onerous by the local partners. Some psychological items caused a feeling of redundancy because of overlap with the questions on emotional distress, which may annoy participants and limit overall data quality [45]. Based on recommendations by the local research team and feedback after the pretest, we selected the 12 items with the highest relevance in this context and smallest overlap (α = 0.76).

### 2.3. Data Analysis

To model the psychosocial consequences of water carrying, we performed generalized estimating equations (GEE), which accounted for the nested structure of the data (individuals nested in communities) [46]. We estimated three separate models for the outcome variables of emotional distress, quality of life, and daily functioning. We included the grand-mean centered physical burden as the predictor, which represents women’s physical burden as compared to the average physical burden (i.e., the typical woman’s burden). For the moderation analyses, we included terrain as three separate, dummy-coded variables (uphill, downhill, both uphill and downhill) and uterine prolapse in two additional GEE models. Carrying on a flat path and not having uterine prolapse were used as reference categories. We adjusted all models for socio-demographic measures (see Table 1), and whether women were currently pregnant or had delivered in the last three months. We adjusted for having uterine prolapse when not included as a moderator. We computed all analyses in IBM SPSS Statistics 24 (IBM Corp., Armonk, N.Y., USA). Sample syntax in SPSS can be found in Supporting Information S3.

## 3. Results

All sociodemographic information on our sample can be found in Table 2. The average observed carrying distance was 81.3 m (*SD* = 162.0 m) with an average weight of 19.4 kg (*SD* = 9.7 kg) and 2.6 trips (*SD* = 2.4) per day. On average, women reported low-to-moderate emotional distress, moderate quality of life, and moderate-to-high daily functioning related to water carrying.

As can be seen in Table S2 in the Supporting Information, GEEs without considering terrain or health indicated that women with higher physical burden of water carrying reported 16% greater emotional distress and 39% lower functioning in daily activities (*B*[*SE*] = 0.16[0.07]; *p* = 0.029; *B*[*SE*] = −0.39[0.09]; *p* < 0.001). The physical burden of water carrying was not related to quality of life.

### 3.1. Moderation by Terrain

There were multiple interaction effects for physical burden and terrain, see Table 3. The main effects in Table 3 indicate the terrain comparisons for women carrying water with average physical burden. Among those women, those carrying on a *flat path* showed no differences in emotional distress or functioning related to physical burden compared to those carrying in hilly areas (see Table 3), although they showed 17% increased quality of life (*p* = 0.029). Women carrying with average physical burden *uphill* reported only slightly lower quality of life (2%, *p* < 0.001) and daily functioning (5%, *p* < 0.001) compared to women walking on a flat path. Similarly, women carrying with average physical burden walking *downhill* reported 4% greater emotional distress (*p* < 0.001) and 9% lower functioning (*p* < 0.001), compared to those carrying on a flat path.

### 3.2. Moderation by Uterine Prolapse

However, of the women carrying with above-average physical burden, those who carried *uphill* reported 33% greater emotional distress (*p* = 0.019) and 25% lower quality of life (*p* = 0.006) compared to those walking on a flat path (moderation effect). Women with above-average physical burden reported 16% lower quality of life (*p* = 0.001) but 64% higher functioning (*p* = 0.008) when carrying *downhill* compared to walking on a flat path. Women with above-average physical burden carrying *both uphill and downhill* reported 30% greater emotional distress (*p* = 0.017) compared to women walking on a flat path.

There were significant interactions of physical burden and uterine prolapse for emotional distress and quality of life, but not for daily functioning, see Table 4. Women with uterine prolapse and average physical burden reported 11% greater emotional distress (*p* < 0.001), 6% lower quality of life (*p* < 0.001), and 7% lower functioning in daily activities (*p* < 0.001) compared to women without uterine prolapse. Women with uterine prolapse and above-average physical burden reported 19% more emotional distress (*p* = 0.028) and 14% higher quality of life (*p* < 0.001) compared to women without uterine prolapse.

## 4. Discussion

The present study indicates how the physical burden of water carrying relates to psychosocial well-being. It highlights that context-specific factors may potentially exacerbate this relationship. In line with our hypotheses, our results indicated that hilly terrain and uterine prolapse aggravate adverse psychosocial consequences of water carrying.

The physical burden of water carrying is directly related to women’s higher emotional distress and reduced functioning in other daily activities besides water carrying. Due to its undeniable necessity, carrying water is an everyday work task [10,11,29]. Our results are in line with those from high-income populations that showed that high chronic physical burden in the working environment adversely affects functioning and emotional distress [20,21], especially with challenging environmental demands and low resources [48].

Interestingly, quality of life was not related to the physical burden of water carrying. Quality of life represents a more general concept of well-being including physical, social, and environmental aspects [38], whereas emotional distress measures the psychological state specifically [44]. The reason why physical burden was not directly related to quality of life may be that many other individual and contextual factors can influence quality of life [49,50].

Our moderation analyses indicated that the relationship between physical burden and psychosocial well-being depended on terrain and personal health. Even greater emotional distress was observed for women who carried heavy loads uphill, or uphill and downhill. While the physical burden of water carrying was not related to quality of life, a relation was observed between the terrain and quality of life: lower quality of life occurs among those who carry uphill or downhill. Interestingly, higher functioning in daily activities was found for women carrying downhill. These findings align with previous studies that showed that certain terrains can put an added risk to physical health [7]. Beyond previous results, our study showed that terrain also moderates the relationship between physical burden and psychosocial well-being. Future studies might explore whether further characteristics of the physical environment (e.g., poor roads or weather conditions [7]) or other contextual factors when retrieving water, such as violence, sexual assault, or dangerous animals, might increase the adverse impact of water carrying on psychological well-being [13,15].

Women with uterine prolapse reported lower psychosocial well-being which is consistent with previous research [29,30]. More importantly, consistent with our hypothesis, uterine prolapse acted as a moderator of the relationship between the physical burden of water carrying and psychosocial well-being. Physical burden related to greater emotional distress for women with uterine prolapse than those without. Since families often depend on women’s ability to work in terms of providing water and food [28,51,52], women may still need to collect water even if affected by uterine prolapse [28,32]. This responsibility possibly adds to the already great psychosocial burden of women with uterine prolapse [31]. Considering that uterine prolapse is also a physical health consequence of frequent water carrying [27], the fact that it additionally seems to exacerbate the emotional burden of water carrying is alarming. Due to the symptoms of uterine prolapse, such as difficulty and pain while walking and lifting, the work performance is not only reduced but also handicapped, which can relate to greater emotional distress [28,29].

Interestingly, for women with uterine prolapse, higher physical burden of water carrying was related to higher quality of life. This result can be an indicator that women who are still able to perform a high physical workload despite suffering from uterine prolapse are likely to be less affected in other areas of life, e.g., economic activities and family life [28,31,32]. This sublines that having access to a close water source is particularly relevant for vulnerable groups. Improvements in infrastructure can support women with uterine prolapse in completing their usual working routine to maintain their social and economic role, improve their quality of life, and reduce emotional distress. For future research, we suggest to also consider other prevalent health conditions, e.g., spinal axial compression, which is more prevalent in African countries due to head-carrying [6] as a moderator on how stressful women perceive the physical workload of water carrying.

### Strengths, Limitations, and Future Directions

The present study is the first to use observed physical variables related to water carrying, e.g., weight and carrying distance, to study its relation to psychosocial well-being. As a limitation, we conducted the study shortly after a rainy season, which may have led to an underestimation of carrying distances and physical burden as many women use farther sources during dry season [35]. Future studies should aim to observe water carrying during both seasons. As a limitation in the measurement, we did not use all items of the quality of life scale to prevent redundancy with questions on emotional distress. This variable may therefore not cover all aspects of quality of life.

The cross-sectional nature of our data does not allow causal conclusions. Future randomized and controlled trials may investigate whether the reduction in physical burden can increase women’s psychosocial well-being.

## 5. Conclusions

Overall, the findings of the association between the physical burden of water carrying and psychosocial well-being bring a new perspective to health research related to water access. They demonstrate not only the complexity, but also the multiple impacts in life that water provision can have for women, and how this interacts with environmental and health factors. Our results underline the interconnectedness of the Sustainable Development Goal (SDG) 6: access to safe water, SDG 3: ensure healthy lives, and SDG 5: gender equality [53].

This study highlights the importance of adequate access to water for women to prevent health impacts such as uterine prolapse and facilitate the quality of life of those already affected. Improvement in the water supply infrastructure, promotion of intermediary solutions such as carts, bicycles, and self-supply options [54], especially for women living in hilly areas, or interventions on behavioural changes, e.g., respecting a water load limit, can hopefully reduce the physical burden of water carrying [55].

## Figures and Tables

**Table 1:** Sample items and descriptive statistics.

Concept	Items	*M*/*f*	*SD*/*f*%
Objective physical burden			
Distance ^1^	Enter distance between household and water source (meters)	81.3	162.0
Weight (kg, sum) ^2,3^	Please indicate the number of different types of containers being carried: 30 L Gagri/Plastic bucket; 20 L Gagri/Plastic bucket; 10 L Gagri/Plastic bucket; 20 L Plastic bottle; 10 L Plastic bottle; 2–5 L Plastic bottle; others (in liters)	19.4	9.7
Trips per day	How many trips do you conduct per day to your primary drinking water source in rainy season?	2.6	2.4
Other loads carried ^3^, control variable	Approximately how many kilogram on average do you carry per trip?	32.6	14.8
Psychosocial well-being			
Emotional distress [37]	19 items (Cronbach’s Alpha = 0.81), e.g., Have you lost interest in things? 0 = no; 1 = yes (19 items)	0.2	0.2
Quality of life [38]	12 items (Cronbach’s Alpha = 0.76), e.g., How would you rate your quality of life? 0 = very poor to 1 = very good	0.7	0.1
Daily functioning [39]	Please rate the severity by which water carrying reduces your daily functioning (1 = not at all to 0 = very much)	0.8	0.3
Moderators			
Terrain	Do you have to walk uphill or downhill to carry the container filled with water from the primary water source back home?		
	uphill	159	16%
	downhill	77	8%
	uphill and downhill	42	4%
	flat path	723	72%
Uterine prolapse	Based on the examined symptoms, does the study participant have uterine prolapse? (% yes)	113	11%

Note: *n* = 1001, *M* = Mean, *SD* = Standard deviation, *f* = frequency, *f*% = relative frequency. All continuous items were recoded to a range between 0 to 1; ^1^ *n* = 980, *n* = 21 missing; ^2^ *n* = 996, *n* = 5 excluded (no regular carrying) ^3^ Outliers < 3 *SD* were adjusted to *M* + 3 *SD* [40].

**Table 2:** Sociodemographic data.

Concept	Items	*M*/*f*	*SD*/*f*%
Age		33.7	9.0
Socioeconomic status ^1^		0.5	0.1
	What kind of fuel do you use MAINLY for cooking?		
	Wood (= 0)	629	63%
	Gas (= 1)	372	37%
	What is the average expenditure of your family per month?		
	Less than 2400 Nepali Rupees (~20 US$)	139	14%
	2500 to 4800 Nepali Rupees (~40 US$)	104	10%
	4900 to 9600 Nepali Rupees (~80 US$)	198	20%
	9700 to 24,000 Nepali Rupees (~200 US$)	443	44%
	>25,000 Nepali Rupees (~208 US$)	117	12%
	Are you the owner of your house? (yes = 1)	980	98%
	How much land does your family own?	56.1	75.0
	How many rooms does your house have?	2.9	1.5
	Does anyone from your household own any of these items? Radio, TV, solar panel, mobile phone, bicycle, motor bike, car, fridge, watch (sum)	3.3	1.3
Education	Illiterate	180	18%
	Informal education	262	26%
	Pre-primary	51	5%
	Primary passed	145	15%
	Lower secondary passed	101	10%
	Secondary	123	12%
	Higher secondary and above	139	14%
Currently pregnant	1 = yes	41	4%
Delivered in last 3 months	1 = yes	24	2%
Ethnicity	Brahmin	304	30%
	Tamang	304	30%
	Newar	60	6%
	Chhetri	59	6%
	Dalit	121	12%
	Rai and Limbu	139	13%
	Others	14	1%

Note: *n* = 1001, *M* = Mean, *SD* = Standard deviation, *f* = frequency, *f*% = relative frequency. Sociodemographic data were used as control variables in all analyses. ^1^ An index (0.0–1.0) was calculated using principle component analysis [47].

**Table 3:** Generalized estimating equations of objective physical burden of carrying water and psychosocial well-being (emotional distress, quality of life, and daily functioning) and its moderation by the terrain.

	Emotional Distress					Quality of Life					Functioning in Daily Activities				
			95% CI					95% CI					95% CI		
	*Estimate*	*SE*	LL	U L	*p*	*Estimate*	*SE*	LL	U L	*p*	*Estimate*	*SE*	LL	U L	*p*
Intercept	0.37	0.07	0.22	0.51	<0.001	0.60	0.03	0.54	0.66	<0.001	0.92	0.07	0.78	1.06	<0.001
Physical burden (carrying on flat path ^1^)	−0.01	0.04	−0.09	0.07	0.786	0.17	0.08	0.02	0.32	0.029	−0.33	0.17	−0.66	<0.01	0.053
Carrying uphill	0.03	0.02	<0.01	0.07	0.052	−0.02	<0.01	−0.03	−0.01	<0.001	−0.05	0.01	−0.06	−0.04	<0.001
Carrying downhill	0.04	0.01	0.03	0.05	<0.001	<0.01	0.01	−0.02	0.01	0.635	−0.09	0.02	−0.14	−0.04	<0.001
Carrying uphill and downhill	0.01	0.02	−0.02	0.05	0.459	−0.02	0.01	−0.05	0.01	0.119	−0.10	0.04	−0.17	−0.03	0.003
Physical burden *Carrying uphill	0.33	0.14	0.06	0.61	0.019	−0.25	0.09	−0.43	−0.07	0.006	−0.22	0.17	−0.55	0.11	0.189
Physical burden *Carrying downhill	−0.08	0.10	−0.28	0.11	0.409	−0.16	0.05	−0.27	−0.06	0.001	0.64	0.24	0.17	1.11	0.008
Physical burden *Carrying uphill and downhill	0.30	0.13	0.06	0.55	0.017	<0.01	0.06	−0.11	0.12	0.980	−0.12	0.15	−0.43	0.18	0.428
Age	<0.01	<0.01	<0.01	<0.01	0.973	<0.01	<0.01	<0.01	<0.01	0.699	<0.01	<0.01	<0.01	<0.01	0.414
Education ^2^	−0.01	<0.01	−0.02	<0.01	0.19	0.01	<0.01	0.01	0.02	<0.001	<0.01	<0.01	−0.01	0.01	0.728
Socio-economic status ^3^	<0.01	<0.01	<0.01	<0.01	<0.001	0.10	0.03	0.05	0.15	<0.001	0.21	0.10	0.01	0.40	0.041
Currently pregnant	<0.01	0.02	−0.03	0.03	0.994	0.03	0.02	−0.01	0.07	0.120	−0.06	0.02	−0.10	−0.01	0.017
Delivered in last 3 months	0.01	0.04	−0.07	0.09	0.830	<0.01	0.03	−0.07	0.06	0.888	<0.01	0.05	−0.09	0.10	0.951
Other heavy loads carried (in kg)	−0.01	0.02	−0.05	0.02	0.496	0.01	0.02	−0.03	0.05	0.510	0.01	0.10	−0.18	0.21	0.896
Uterine prolapse	0.12	0.02	0.07	0.16	<0.001	−0.05	<0.01	−0.06	−0.05	<0.001	−0.06	0.01	−0.08	−0.03	<0.001
Ethnicity ^4^															
Brahmin	0.03	0.01	<0.01	0.05	0.057	−0.02	0.01	−0.04	0.01	0.145	−0.14	0.01	−0.16	−0.13	<0.001
Tamang	−0.03	0.01	−0.05	−0.01	0.001	−0.02	0.01	−0.04	−0.01	0.010	−0.16	0.01	−0.19	−0.13	<0.001
Newar	0.02	0.01	−0.01	0.04	0.166	<0.01	0.01	−0.02	0.02	0.931	−0.14	0.03	−0.19	−0.09	<0.001
Chhetri	0.02	0.02	−0.02	0.05	0.324	−0.01	0.02	−0.05	0.04	0.739	−0.16	0.06	−0.27	−0.05	0.003
Dalit	0.07	0.02	0.02	0.11	0.008	−0.01	0.01	−0.02	<0.01	0.156	−0.08	0.02	−0.11	−0.04	<0.001
Rai and Limbu	−0.02	0.01	−0.04	<0.01	0.020	−0.01	0.01	−0.04	0.01	0.330	−0.06	0.01	−0.08	−0.03	<0.001

Note: *n* = 978 (*n* = 21 distance missing, *n* = 2 information on uterine prolapse missing). Five communities. *Estimate* = Parameter Estimates. *SE* = Standard Error. CI = Confidence interval. Probability distribution: normal, link function: identity. All p-values are two-tailed. ^1^ Reference category. ^2^ Higher values refer to a higher level of education: 0 = Illiterate, 1 = Informal education, 2 = Pre-primary, 3 = Primary passed 4 = Lower secondary passed, 5 = Secondary, 6 = Higher secondary and above. ^3^ An index (0.0–1.0) was calculated using principle component analysis [47]. ^4^ Reference category = other. * indicates the interaction effect (moderator).

**Table 4:** Generalized estimating equations of objective physical burden of carrying water and psychosocial well-being and its moderation by uterine prolapse.

	Emotional Distress					Quality of Life					Functioning in Daily Activities				
			95% CI					95% CI					95% CI		
	*Estimate*	*SE*	LL	UL	*p*	*Estimate*	*SE*	LL	UL	*p*	*Estimate*	*SE*	LL	UL	*p*
Intercept	0.39	0.07	0.25	0.53	<0.001	0.57	0.03	0.52	0.63	<0.001	0.85	0.08	0.68	1.01	<0.001
Physical burden (without uterine prolapse ^1^)	0.07	0.06	−0.06	0.19	0.293	0.04	0.06	−0.09	0.16	0.577	−0.42	0.11	−0.64	−0.20	<0.001
Uterine prolapse	0.11	0.02	0.07	0.15	<0.001	−0.06	0.01	−0.07	−0.05	<0.001	−0.07	0.02	−0.11	−0.04	<0.001
Physical burden *uterine prolapse	0.19	0.09	0.02	0.36	0.028	0.14	0.04	0.06	0.22	<0.001	0.23	0.13	−0.03	0.49	0.077

*Note: n =* 978 (*n* = 21 distance missing, *n* = 2 information on uterine prolapse missing). Five communities. *Estimate =* Parameter Estimates. *SE* = Standard Error. CI = Confidence interval. Probability distribution: normal, link function: identity. All *p*-values are two-tailed. ^1^ Reference category. These results were adjusted for the same sociodemographic variables as displayed in Table 3 but not reported in this table. * Interaction term as this is a moderation analysis.

## Data Availability

The data is available from V.M.J.T at request.

## Supplementary Materials

The following are available online at https://www.mdpi.com/article/10.3390/ijerph18157908/s1, Table S1: Items. Table S2: Generalized estimating equations of objective physical burden of carrying water and psychosocial well-being (emotional distress, quality of life, and daily functioning). S3: Sample Syntax for SPSS.
