# Supplementary material for: The Physical Burden of Water Carrying and Women’s Psychosocial Well-Being: Evidence from Rural Nepal
Source: Int J Environ Res Public Health. 2021 Jul 26;18(15):7908. doi: 10.3390/ijerph18157908 (PMC8345449; doi:10.3390/ijerph18157908)
Supplement: Supplementary file 1 [file ijerph-18-07908-s001.zip › ijerph-1282677-supplementary.pdf]

The physical burden of water carrying and women's psychosocial well-being: Evidence from rural Nepal

SUPPLEMENTARY MATERIAL

Vica Marie Jelena Tomberge [1,*,†], Janine Stefanie Bischof [1,†], Regula Meierhofer [2], Akina Shrestha [3] and Jennifer Inauen [1]

[1] Department of Health Psychology & Behavioral Medicine, Institute of Psychology, University of Bern,
Fabrikstrasse 8, 3012 Bern, Switzerland; vica.tomberge@psy.unibe.ch (V.M.J.T); janine.bischof@psy.unibe.ch (J.S.B.); jennifer.inauen@psy.unibe.ch (J.I.)
[2] Eawag, Swiss Federal Institute of Aquatic Science and Technology, Department of Sanitation, Water and Solid Waste for Development (Sandec), Ueberlandstrasse 133, 8600 Duebendorf, Switzerland; Regula.Meierhofer@eawag.ch
[3] School of Medical Sciences, Dhulikhel Hospital, Kathmandu University Hospital, 1008 Dhulikhel, Nepal; akinakoju@gmail.com
* Correspondence: vica.tomberge@psy.unibe.ch
† These authors contributed equally and share first authorship.

Table S1. Items...1

Table S2. Generalized estimating equations of objective physical burden of carrying water and psychosocial well-being (emotional distress, quality of life, and daily functioning). 3

S3. Sample Syntax for SPSS..4

Table S1. Items.

Concept		Items
Physical burden (1)[1,2]		
	Weight	Please indicate the number of different types of containers being carried. *30l Gagri/Plastic bucket; 20l Gagri/Plastic bucket; 10l Gagri/Plastic bucket; 20 l Plastic bottle; 10 l Plastic bottle; 2-5 l Plastic bottle; others (in liters)*
	Distance	Enter distance between household and water source (meters)
	Frequency	How many trips do you conduct per day to your primary drinking water source in rainy season?
Emotional distress (2)		Do you often have headaches? *0 = no; 1 = yes*
		Is your appetite poor? *0 = no; 1 = yes*
		Do you sleep badly? *0 = no; 1 = yes*
		Are you easily frightened? *0 = no; 1 = yes*
		Do your hands shake? *0 = no; 1 = yes*
		Do you feel nervous, tense or worried? *0 = no; 1 = yes*
		Is your digestion poor? *0 = no; 1 = yes*
		Do you have trouble thinking clearly? *0 = no; 1 = yes*
		Do you feel unhappy? *0 = no; 1 = yes*
		Do you cry more than usual? *0 = no; 1 = yes*
		Do you find it difficult to make decisions? *0 = no; 1 = yes*
		Is your daily work suffering? *0 = no; 1 = yes*
		Are you unable to play a useful part in life? *0 = no; 1 = yes s*
		Have you lost interest in things? *0 = no; 1 = yes*
		Do you feel you are a worthless person? *0 = no; 1 = yes*
		Has the thought of ending your life been on your mind? *0 = no; 1 = yes*
		Do you have uncomfortable feelings in your stomach? *0 = no; 1 = yes*
		Are you easily tired? *0 = no; 1 = yes*
		Do you find it difficult to enjoy your daily activities? *0 = no; 1 = yes*

Note: Gagri is a traditional water pot in Nepal. [1]Calculated according to recommendations on calculating risk assessment for lifting and carrying suggested by the Swiss National Accident Insurance Fund (1). [2]All items used a five-point Likert scale and were recoded to a range between 0 to 1; *0 = not at all*, 0.25 = *somewhat* 0.5 = *rather* 0.75 = *quite 1 = very much.*

Quality of Life[2](3)	How would you rate your quality of life? *0 = very poor to 1 = very good*<br><br>How satisfied are you with your health? *0 = not satisfied at all to 1 = very satisfied*<br><br>To what extent do you feel that physical pain prevents you from doing what you need to do? *0 = not at all to 1 = very much*<br><br>How much do you enjoy life? *0 = not at all to 1 = very much*<br><br>How safe do you feel in your daily life? *0 = not safe at all to 1 = very safe*<br><br>Are you able to accept your bodily appearance? *0 = not at all to 1 = very much*<br><br>Have you enough money to meet your needs? *0 = not at all to 1 = very much*<br><br>How well are you able to get around? *0 = not at all to 1 = very much*<br><br>How satisfied are you with your personal relationships? *0 = not satisfied at all to 1 = very satisfied*<br><br>How satisfied are you with your sex life? *0 = not satisfied at all to 1 = very satisfied*<br><br>How satisfied are you with the conditions of your living place? *0 = not satisfied at all to 1 = very satisfied*<br><br>How satisfied are you with your access to health services? *0 = not satisfied at all to 1 = very satisfied*
Daily functioning (4) [2]	Please rate the severity by which water carrying reduces your daily functioning<br><br>*0 = not at all to 1 = very much*
Uterine prolapse	Based on the examined symptoms, does the study participant have uterine prolapse? *0 = no; 1 = yes*
Terrain	Do you have to walk uphill or downhill to carry the container filled with water from the primary water source back home during the dry season?<br><br>*1 = uphill; 2 = downhill; 3 = uphill and downhill; 4 = flat*

Table S2. Generalized estimating equations of objective physical burden of carrying water and psychosocial well-being (emotional distress, quality of life, and daily functioning).

	Emotional distress					Quality of life					Functioning in daily activities				
			95% CI					95% CI					95% CI		
	Estimate	*SE*	*LL*	*UL*	*p*	*Estimate*	*SE*	*LL*	*UL*	*p*	*Estimate*	*SE*	*LL*	*UL*	*p*
Intercept	0.36	0.07	0.23	0.49	< 0.001	0.59	0.03	0.53	0.64	< 0.001	0.87	0.08	0.71	1.03	< 0.001
Physical burden	0.16	0.07	0.02	0.30	0.029	0.05	0.06	-0.06	0.16	0.387	-0.39	0.09	-0.56	-0.21	< 0.001
Age	< 0.01	< 0.01	< 0.01	< 0.01	0.700	< 0.01	< 0.01	< 0.01	< 0.01	0.880	< 0.01	< 0.01	< 0.01	< 0.01	0.419
Education[1]	-0.01	0.01	-0.02	< 0.01	0.135	0.01	< 0.01	0.01	0.02	< 0.001	< 0.01	< 0.01	< 0.01	0.01	0.337
Socio-economic status[2]	-0.24	0.06	-0.36	-0.12	< 0.001	0.10	0.03	0.04	0.15	0.001	0.19	0.11	-0.03	0.41	0.085
Currently pregnant	< 0.01	0.02	-0.04	0.04	0.912	0.03	0.02	-0.01	0.08	0.113	-0.06	0.02	-0.10	-0.01	0.014
Delivered in last 3 months	0.01	0.04	-0.07	0.10	0.767	< 0.01	0.03	-0.07	0.06	0.890	0.01	0.05	-0.10	0.11	0.920
Other heavy loads carried (in kg)	-0.01	0.01	-0.04	0.01	0.343	0.01	0.02	-0.03	0.05	0.604	0.02	0.10	-0.18	0.21	0.881
Ethnicity[3]															
Brahmin	0.02	0.01	< 0.01	0.04	0.106	-0.01	0.01	-0.03	0.02	0.492	-0.12	0.02	-0.15	-0.09	< 0.001
Tamang	-0.04	0.01	-0.06	-0.01	0.003	-0.01	0.01	-0.03	< 0.01	0.074	-0.14	0.02	-0.17	-0.10	< 0.001
Newar	0.01	0.01	-0.01	0.03	0.203	0.01	0.01	-0.01	0.02	0.413	-0.12	0.02	-0.16	-0.09	< 0.001
Chhetri	0.01	0.02	-0.03	0.05	0.744	< 0.01	0.02	-0.04	0.04	0.924	-0.14	0.06	-0.25	-0.02	0.026
Dalit	0.06	0.02	0.03	0.10	0.001	< 0.01	0.01	-0.02	0.02	0.732	-0.05	0.02	-0.09	-0.01	0.021
Rai and Limbu	-0.02	0.01	-0.05	< 0.01	0.093	-0.01	0.01	-0.03	0.02	0.638	-0.03	0.03	-0.08	0.02	0.200

Note: N= 980 (*n* = 21 distance missing). 5 communities. *Estimate* = Parameter Estimates. *SE* = Standard Error. CI = Confidence interval. Probability distribution: normal, link function: identity. All *p*-values are two-tailed. [1]Higher values refer to a higher level of education: 0 = Illiterate , 1 = Informal education, 2 = Pre-primary, 3 = Primary passed 4= Lower secondary passed, 5= Secondary, 6 = Higher secondary and above. [2]An index (0.0-1.0) was calculated using principle component analysis (5). [3]Reference = other

S3. Sample Syntax for SPSS.

**Calculating physical burden according to an adapted version of the risk assessment for lifting and carrying suggested by SUVA, the Swiss National Accident Insurance Fund (1)

** The adapted formula includes the following weighted risk variables: (Weight + environmental condition)*(carrying frequency*distance).

```
RECODE weight (Lowest thru 4.9999=1) (5 thru 9.999=2) (10 thru 14.999=4)
    (25 thru Highest=25) (15 thru 24.999=7) INTO weight_category.
VARIABLE LABELS  weight_category 'suva category weight'.
EXECUTE.


RECODE distance_cor (Lowest thru 299.9999=1) (300 thru 999.99=2) (1000 thru Highest=4)
    INTO distance_category.
VARIABLE LABELS  distance_category 'suva category distance'.
EXECUTE.


COMPUTE suva_o= (trips_rainyseason_all*distance_category) * (weight_category+1).
VARIABLE LABELS  suva_o 'suva risk evaluation without body posture'.
EXECUTE.


**Recoding to 0-1 scale
DESCRIPTIVES VARIABLES=suva_o
  /STATISTICS=MEAN STDDEV MIN MAX.
COMPUTE suva_o_r=(suva_o-2)/(416-2).
EXECUTE.


**Grand-mean centering
DESCRIPTIVES VARIABLES=suva_o_r
  /STATISTICS=MEAN .
COMPUTE suva_o_GM=  suva_o_r- 0.0653.


 *** Example to calculate main effect (emotional distress) in GEE
GENLIN  em_dis (REFERENCE=FIRST) WITH suva_o_GM age
```

```
education SI_SES curr_pregnant delivered kilos_day_all_wins_trans  by eth

  /MODEL suva_o_GM age education SI_SES curr_pregnant delivered kilos_day_all_wins_trans eth

 INTERCEPT=YES

 DISTRIBUTION=NORMAL LINK=IDENTITY

  /CRITERIA METHOD=FISHER(1) SCALE=1 MAXITERATIONS=100 MAXSTEPHALVING=5 PCONVERGE=1E-006(ABSOLUTE)

    SINGULAR=1E-012 ANALYSISTYPE=3(WALD) CILEVEL=95 LIKELIHOOD=FULL

  /REPEATED SUBJECT=vdc_municipality WITHINSUBJECT=Participant_ID SORT=YES CORRTYPE=EXCHANGEABLE ADJUSTCORR=YES

    COVB=ROBUST MAXITERATIONS=100 PCONVERGE=1e-006(ABSOLUTE) UPDATECORR=1

  /MISSING CLASSMISSING=EXCLUDE

  /PRINT CPS DESCRIPTIVES MODELINFO FIT SUMMARY SOLUTION (EXPONENTIATED) COVB.


*** Example to calculate moderation effect (uterine prolapse on emotional distress) in GEE

GENLIN  em_dis (REFERENCE=FIRST) WITH suva_o_GM age

education SI_SES curr_pregnant delivered kilos_day_all_wins_trans uterus_prolapse by eth

  /MODEL suva_o_GM suva_o_GM*uterus_prolapse uterus_prolapse age education SI_SES curr_pregnant delivered kilos_day_all_wins_trans  eth

 INTERCEPT=YES

 DISTRIBUTION=NORMAL LINK=IDENTITY

  /CRITERIA METHOD=FISHER(1) SCALE=1 MAXITERATIONS=100 MAXSTEPHALVING=5 PCONVERGE=1E-006(ABSOLUTE)

    SINGULAR=1E-012 ANALYSISTYPE=3(WALD) CILEVEL=95 LIKELIHOOD=FULL

  /REPEATED SUBJECT=vdc_municipality WITHINSUBJECT=Participant_ID SORT=YES CORRTYPE=EXCHANGEABLE ADJUSTCORR=YES

    COVB=ROBUST MAXITERATIONS=100 PCONVERGE=1e-006(ABSOLUTE) UPDATECORR=1

  /MISSING CLASSMISSING=EXCLUDE

  /PRINT CPS DESCRIPTIVES MODELINFO FIT SUMMARY SOLUTION (EXPONENTIATED) COVB.
```

1. SUVA, Swiss National Accident Insurance Fund, editor. Gefährdungsermittlung: Heben und Tragen [risk assessment for lifting and carrying of the SUVA, Swiss National Accident Insurance Fund] 2019. Available online: https://www.suva.ch/de-CH/material/Dokumentationen/beurteilung-der-koerperlichen-belastung-heben-und-tragen-von-lasten-88190d1812918129 (accessed on 9 March 2021)

2. Youngmann R, Zilber N, Workneh F, Giel R. Adapting the SRQ for Ethiopian Populations: A Culturally-Sensitive Psychiatric Screening Instrument. Transcult Psychiatry. 2008 Dec;45(4):566–89.

3. World Health Organization, editor. Development of the World Health Organization WHOQOL-BREF quality of life assessment. Psychol Med. 1998;23(3):551–8.

4. de Jong K, Ariti C, van der Kam S, Mooren T, Shanks L, Pintaldi G, et al. Monitoring and Evaluating Psychosocial Intervention Outcomes in Humanitarian Aid. Faragher EB, editor. PLOS ONE. 2016 Jun 17;11(6):e0157474.

5. Krishnan V. Constructing an Area-based Socioeconomic Index: A Principal Components Analysis Approach. Early Child Development Mapping Project; 2010.
